# Implementation of new guidelines in the prehospital services: a nationwide survey of Norway

**DOI:** 10.1186/s13049-019-0660-0

**Published:** 2019-08-29

**Authors:** Nina Øye Thorvaldsen, Lars Didrik Flingtorp, Torben Wisborg, Elisabeth Jeppesen

**Affiliations:** 1 Emergency Medical Services, Division of Prehospital Services, Oslo University Hospital, Oslo, Norway; 2 Norwegian National Advisory Unit on Prehospital Emergency Medicine (NAKOS), Oslo, Norway; 3 Norwegian National Advisory Unit on Trauma, Division of Emergencies and Critical Care, Oslo University Hospital, Oslo, Norway; 4 Anesthesia and Critical Care Research Group, Faculty of Health Sciences, University of Tromsø – the Arctic University of Norway, Tromsø, Norway; 5 Faculty of Health Science, University of Stavanger, Stavanger, Norway

**Keywords:** Emergency medical services, Guideline, Implementation, Prehospital, Rigid cervical collar, Spinal immobilization, Spinal stabilization

## Abstract

**Background:**

A debate regarding the potential harmful effects of rigid neck collar and backboard usage among prehospital and hospital care providers in Norway provoked the development of an evidence-based guideline. “The Norwegian guideline for the prehospital management of adult trauma patients with potential spinal injury” was developed with rigorous scientific methods and published in 2016. An e-learning course was developed in parallel. The aim of this study is to explore whether emergency medical services personnel in Norway have implemented the guideline, and to what extent the e-learning course was applied during the implementation process.

**Method:**

An electronic survey was distributed individually to registered prehospital personnel in Norway 18 months after publication of the guideline.

**Results:**

In all, 938 of 5500 (17%) EMS personnel responded to the survey. More than one-half confirmed knowledge of the guideline; among these, 56% claimed that the guideline was implemented in the service they work. Not having responded to trauma cases in real life was the main reason for not having executed the guideline. The e-learning course had been completed by 18% of respondents.

**Conclusion:**

Although the guideline has not been authorized or made compulsory by national authorities, one-half of respondents with knowledge of the guideline reported it as implemented. E-learning did not seem to have affected the implementation. The guideline was developed based on perceived needs among care providers, and this probably facilitated implementation of the guideline.

**Electronic supplementary material:**

The online version of this article (10.1186/s13049-019-0660-0) contains supplementary material, which is available to authorized users.

## Background

Although spinal cord injury caused by trauma is uncommon, it can result in severe consequences for patients [1]. Of 98,200 admitted trauma patients across Europe between 2014 and 2016, 29,653 (30.2%) suffered spine (including neck) injury [2]. By comparison, 1549 (24%) of 6375 trauma patients admitted to hospital in Norway during 2016 suffered from spinal injury [3].

Treatment of trauma patients with a potential spinal (column and cord) injury have been focused on preventing secondary injuries to a potentially unstable spine. Patients exposed to certain mechanisms of injury have been immobilized, strapped to a rigid backboard in the supine position with a cervical collar in place, regardless of clinical complaints [4]. Those principles were introduced with the Prehospital Trauma Life Support (PHTLS) guidelines [5] and have dominated the field of prehospital trauma care for decades. Nevertheless, focus has shifted towards possible negative effects caused by immobilizing patients, particularly a possible increase in intracranial pressure by applying a rigid neck collar to patients with a traumatic brain injury [6–8] and reduced airway patency caused by being strapped on a rigid spine board in the supine position [9]. These potential negative consequences resulted in divergent procedures for prehospital management of this patient group in Norway. There are currently 18 emergency medical services (EMS) in Norway with individual medical directors and no guarantee of consistency or national cooperation. The services adhere to a variety of local guidelines, procedures and manuals [10, 11], which has resulted in a discrepancy in how trauma patients are treated.

As a result of this discrepancy in pre-hospital treatment of patients with potentially unstable spine injuries, the Norwegian National Advisory Unit on Trauma (NKT-T) in collaboration with The Norwegian Knowledge Centre for the Health Services (NOKC), both governmental national organs, commissioned a multi-disciplinary group to provide a collective guideline on this topic [12]. Evidence-based guidelines are recommendations based on a systematic review of the available literature, with the intention to reduce inappropriate variation and improve care and patient outcome by transferring evidence to practice [13]. (Inter) national guidelines should result in similar treatment independent of patient location. In order to develop this national guideline, a systematic review of available literature on this topic was conducted and the Grading of Recommendations Assessment, Development, and Evaluation (GRADE) framework was used to determine the quality of the evidence [14]. The guideline was developed based upon the Appraisal of Guidelines for Research & Evaluation (AGREE) instrument. The AGREE instrument is a “…tool that assesses the methodological rigor and transparency in which a guideline is developed...” and it is used internationally [15]. Because evidence on the topic is limited, a standardized consensus process was used to help inform the guideline.

**Fig. 1:**
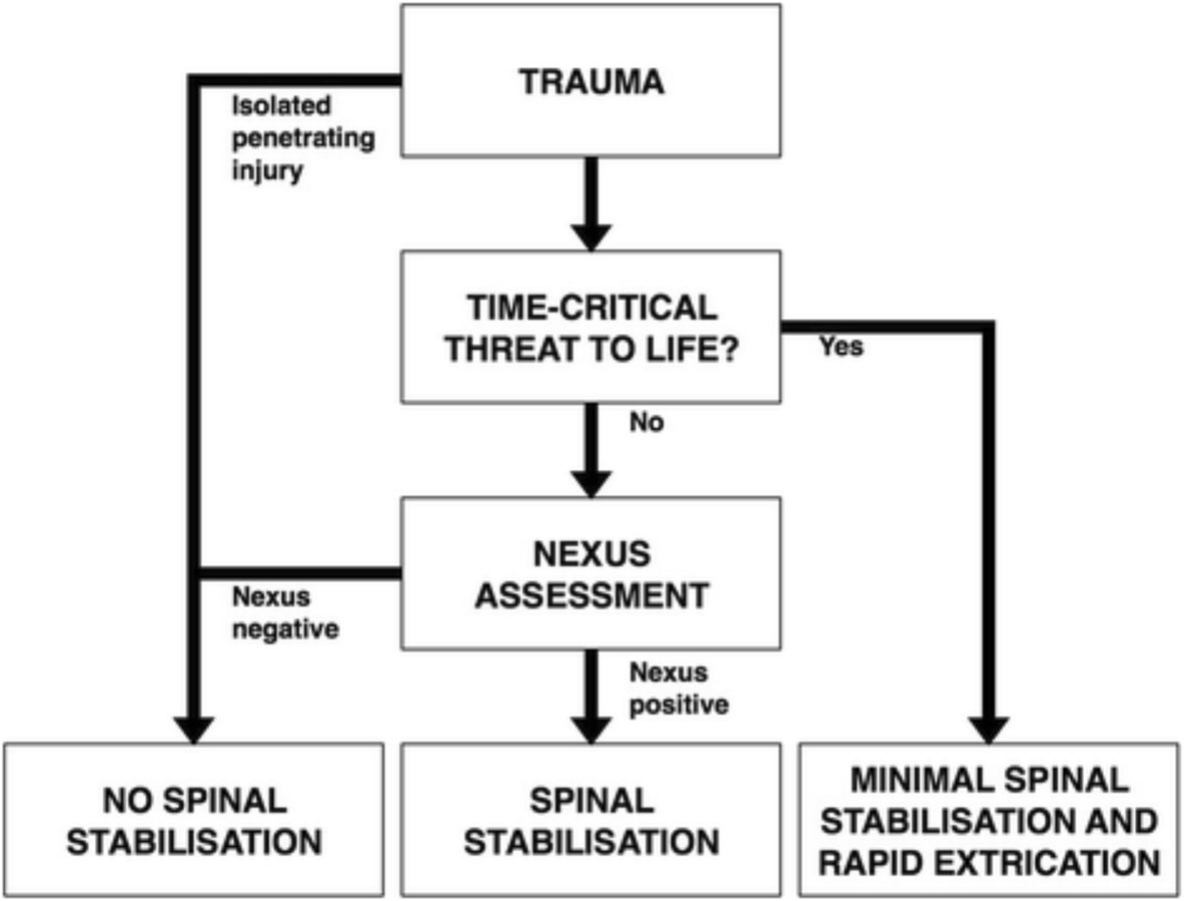
Flowchart describing pre-hospital spinal stabilization in patients with suspected spinal injury from the guideline, as published in English, with permission from the copyright holder [12]. Nexus: National Emergency X-Radiography Utilization Study [19]

The guideline, together with a flowchart (Fig. 1), was published in 2016 with open access at NOKC web pages, which permits unrestricted use [16]. This database is national, containing guidelines and procedures approved by at least one health trust in order to allow other health trusts to approve them for local use and subscribe to revisions. The guideline was presented through passive dissemination; it was introduced at national and international conferences and published in the Scandinavian Journal of Trauma, Resuscitation and Emergency Medicine [12]. Guidelines developed by the Directorate of Health in Norway are normative [17]. Other guidelines must be approved by the senior individual responsible for guidelines in each EMS to become a national standard. To contribute to the implementation process, an e-learning course was developed by the Norwegian National Advisory Unit on Prehospital Emergency Medicine (NAKOS). It was made available to all prehospital personnel at the NAKOS web page for registered users December 2016 [18].

Future evidence-based guidelines developed and intended for national use will hopefully benefit from the experiences of this implementation process. The aim of this study was to explore whether EMS in Norway had implemented the guideline at the level of the individual practitioner, and to what extent the e-learning course “Stabilizing the spine” at the NAKOS web page had been applied during the process.

## Methods

### Setting

The four Regional Health Trusts are responsible for providing EMS within their region [20]. The emergency medical chain consists of 1) emergency medical communication centers organized by regional health trusts; 2) primary care physicians on-call; and 3) EMS consisting of car-, boat-, and air ambulance. The air ambulance helicopters are staffed with an anesthesiologist, and air ambulance fixed-wing airplanes are staffed with nursing specialists (anesthesia or intensive care) [21]. The car and boat ambulances are staffed with emergency medical technicians and/or paramedics. The resources are dispatched by the emergency medical communication centers, which use the Norwegian Index of Medical Emergencies as a decision tool to determine level of emergency and what resources to dispatch [22].

### Data collection

An electronic questionnaire with 15 questions regarding implementation of the guideline was distributed by personal email to all prehospital personnel registered in the NAKOS web portal [18] (Additional file 1). The portal provides e-learning courses serving the purpose of introduction and training. The NAKOS portal served as an important source of education during the recent inception of the national guideline for emergency agencies regarding ongoing life-threatening violence [23] and the new national emergency communication system [24]. Therefore, all prehospital personnel are assumed to have a user account in the NAKOS web portal some more than one account due to change of employer etc. More than 7000 emails were sent. Statistics Norway reported 5550 persons employed in the EMS in Norway in 2017 [25, 26]. For the purposes of the study, this population included persons performing at least 1 h of income-producing work during on the week or day referred to, as well as persons who have this sort of work, but were temporarily absent due to illness, vacation, paid leave, etc. Persons in the civil service and conscripts were considered employed persons. Individuals who were involuntary laid off for a continuous duration of up to 3 months were defined as employed and temporarily absent [27].

The questionnaire was distributed 18 months after the publication of the guideline. The study period lasted from May 2017 to October 2017. The director of the EMS at Oslo University Hospital emailed the other EMS directors in Norway and encouraged them to circulate a reminder of the questionnaire, a premade attachment, after the summer holidays.

The questionnaire was designed in Questback Essentials [28] and transferred to IBM SPSS version 25 [29]. A pilot study was distributed to five EMS personnel in advance and the questionnaire was modified after feedback. The questionnaire’s design permits the author to guide respondents through the questions based on their answers. For example, those who answered “no” to the question “Do you have knowledge to the guideline …” did not receive any further questions about the guideline.

### Statistics

Frequency distribution and percentage are reported as categorical variables. A χ^2^ test for independence was used to determine association between categorical variables. *P*-value (≤0.05) was used as the threshold for statistical significance.

Two open ended questions were analyzed using a qualitative method inspired by Graneheim and Lundman [30].

Possible answers to the question “Is the procedure, “Stabilizing the spine”, applied in the service where you work?” were “Yes”, “No”, and “I do not know”. The alternatives “No” and “I do not know” were combined into one category.

In an open-ended question, the respondents were asked to describe comments received from the emergency department (ED) personnel when delivering patients assessed using the guideline, and other comments on the guideline.

### Ethics

When generating a user account in the NAKOS portal, users sign a declaration of consent that allows data collected in the portal to be used for research and quality assurance [31]. Data from the present study did not contain personal information, therefore the study did not need to report to The Norwegian Center for Research data [32]. The answers received were anonymous. Questback central is unable to trace originating IP addresses when the data is deleted from the user page; therefore, all data were deleted from the user page after being transferred to SPSS. Because this was a quality assurance project, approval from the Regional Committees for Medical and Health Research Ethics was not necessary according to Norwegian regulations [33].

### Results

An estimated 7000 questionnaires were distributed. Statistics Norway reported 5550 employed persons in the EMS in Norway in 2017 [29], for an estimated response rate of 17% (938 of 5550). Demographic data is shown in Table 1.

**Table 1:** Characteristics of the respondents

	Number (n)	Percentage (%)
Number of respondents	938	
Gender		
Male	625	67
Female	313	33
Age, years		
≤ 24	91	10
25–35	282	30
36–45	285	30
46–59	255	27
≥ 60	25	3
Professional position^a^		
Authorized ambulance emergency medical technician without medical delegations (EMT)	311	33
Authorized ambulance emergency medical technician with medical delegations (paramedic)	546	58
Nurse	140	15
Apprentice	52	6
Supervisor with clinical patient care	38	4
Supervisor without clinical patient care	35	4
Medical doctor	11	1
Rescuer in air ambulance services	7	1
Other	54	6
Clinical experience, years		
0–2	118	13
3–6	196	21
7–15	348	37
≥ 16	279	30

^a^Some respondents reported more than one position

Fifty-three percent of respondents (496 of 938) confirmed they had knowledge of the guideline, and 279 (56% of 496) respondents, representing all 18 services, reported that the guideline was implemented in the service where they work. Furthermore, 43 respondents reported that the guideline was in the process of being implemented in the service where they work. Of those with knowledge of the guideline, 319 (64% of 496) respondents report having used the guideline in real patient situations. The guideline name used in the distributed questionnaire was the name described in the Norwegian guideline [34]. It seems that some EMS have implemented the guideline, but named the guideline differently, which may have led to misinterpretation by the respondents.

No statistically significant difference was found regarding age and knowledge of the guideline. The educational level had no statistically significant influence on reported guideline knowledge.

Respondents who did have knowledge of the guideline, but reported not having executed it (177 of 496) were asked why, and allowed to respond with more than one answer. Eighty-four had not responded to calls where the guideline was needed; 47 had not been properly trained to use the guideline; 45 claimed the guideline had not been approved by their employer; 27 said the written guideline was not available when they were responding to a call; 2 did not understand the guideline; and 25 responded “Other”. No respondents claimed not to have confidence in the guideline.

The majority of respondents with knowledge of the guideline (91% of 496) found the guideline useful when assessing a patient, and the flowchart easy to understand (82% of 496).

### Additional information from open-ended questions

The respondents reported that EMS personnel are frequently asked to justify their assessment and treatment of the patient if the patient arrives in the emergency department (ED) without a rigid neck collar. They also report that rigid neck collars are regularly applied in the ED if they have not been applied by the prehospital personnel.

When asked to comment on the guideline and flowchart, some respondents called for clarification of aspects of the guideline: “why do not patients with penetrating trauma need to be stabilized”, “if the patient has pain in the lumbar area, does he/she need a rigid neck collar” and “wish there were examples”. The majority of respondents found the flowchart easy to understand, but some would like it to be simplified. Several would like to have the flowchart laminated in a pocket-sized version.

### E-learning

Of the 938 respondents, 897 (95.6%) reported that the service they work in uses the NAKOS portal for certifications and courses. However, only 158 (18% out of 897) had completed the e-learning course “Stabilizing the spine”. Of those who had executed the guideline, 118 (37% out of 319) reported to have completed the same e-learning course.

## Discussion

This study assessed implementation of a new evidence-based guideline at an individual practitioner level. Despite the low response rate, the respondents seem to reflect the EMS personnel population in general when considering age, gender, education, employment and years of experience. We found that a guideline developed based on perceived needs by practitioners, using an evidence-based approach and involving professionals from all involved groups, to a large extent was implemented within 18 months. Implementation happened despite the lack of official national endorsement. An accompanying e-learning course did not seem to be influential.

### Implementation strategy

Implementation measures directed at healthcare professionals regarding professional guidelines are shown to have varying effect with modest effect on health outcome [35, 36]. Implementation of new guidelines can be assessed at a service-level, in this case at level of the Health Trust, or at the practitioner level. Official implementation at service level does not automatically imply internalization at practitioner level. With about one-half of the respondents reporting knowledge of the guideline, and approximately one-fourth claiming that the guideline was implemented in the service where they work, the guideline has to some extent been implemented. Passive dissemination as implementation strategy is debated; one critique is that it does not have an effect on patient outcome [35, 37–39]. Publishing the guideline in a national repository for others to adopt was deemed the best option considering there are 18 EMS in Norway whose cooperation has potential for improvement [10]. In this way, the responsibility of approval and further implementation was passed on to those accountable in each EMS. This context considered, passive dissemination had its mission through informing key personnel about the guideline’s existence, a prerequisite for further implementation.

### Hindrance of adoption

#### Lack of approval

Approval by the health trust is a prerequisite for prehospital personnel to adopt and execute the guideline. Only guidelines developed by the Directorate of Health are national, normative, and consequently implemented without the need of local approval [17]. We do not know how the responsible party in each EMS appraised the guideline, but disagreement [36, 40] or a perception of the guideline as being too liberal compared with conventional treatment [41] is known to create resistance. Greater espousal was perhaps anticipated considering that the guideline is published with open access, addresses a controversial topic, and is evidence-based [16]. Evidence-based health services are an ambition internationally [42] and nationally [43]. Development of evidence-based guidelines requires knowledge of the process, which is known to be cost- and time-consuming [36]. Considering this, the guideline could be regarded as a benefit to the EMS, which traditionally each develop their own guidelines [10]. Implementation is known to take time [44]. Several respondents reported the guideline was to be implemented in their service, which indicates that it is likely that further EMS were in the middle of an approval and implementation planning process when the study period closed. Still, the present study does not reveal whether individual practitioners have implemented the guideline personally without official Health Trust endorsement.

#### Training and rate of occurrence

Not having responded to calls where the guideline was needed and not having received enough training were stated as reasons from the respondents that they had not executed the guideline. The car, boat, and air (primary missions only) ambulance executed 708,442 missions during 2016. Considering that 6375 trauma patients were admitted with full trauma team activation in Norway during the same year, the exposure to, and therefore experience with, trauma patients is sparse [3]. Training with a facilitator who understands the guideline is suggested as a means to transfer knowledge to practice. A facilitator can ensure ambiguities are clarified and the guideline is being correctly utilized [45–47]. Simulation has the potential to fill the gap created by limited exposure to and little experience with trauma patients [48]. In a master thesis written by Christiansen (2016), investigating quality assurance in the EMS in Norway, 12 representatives with responsibility for professional development were interviewed about quality assurance in their respective EMS. Simulation or systematic training with or without a facilitator is not used as a method for quality assurance in any of the services. Theoretical and practical tests are primarily used. None of the EMS represented in the master thesis provided systematic feedback to their employees on their work performance [11]. This leaves the impression that the EMS personnel are expected to perform in rare, critical situations as well as quality assurance tests, without any way to learn and prepare other than self-study and self-evaluation. Training is costly and time-consuming considering that employees are taken out of service while preparedness must continue as normal, but education and training are demonstrably important for prehospital personnel’s guideline adherence, as a means to transform theory into practice [49, 50].

#### Interaction with the ED

The respondents were asked to describe what feedback they received when patients assessed according to the guideline were delivered to the ED. Respondents’ descriptions reflect that several EDs operate with guidelines different from the EMS. The treatment policy of the ED receiving a patient can affect the EMS personnel guideline adherence depending on positive or negative feedback [41, 51]. The need for prehospital and in-hospital collaboration is emphasized by the title of the Norwegian guideline, “Stabilizing the spine from the prehospital scene to clarification” (authors translation) [34]. For the sake of the patient and the health care personnel involved, patterns of behavior and treatment should be coordinated by the EMS and EDs.

### E-learning

Considering the number of employees with knowledge of the guideline, few had completed the e-learning course “Stabilizing the spine”. Among those who had executed the guideline, one third had completed the e-learning course. The present study indicates that most EMS personnel are aware of the NAKOS portal. The e-learning courses in the NAKOS portal serve several purposes:

- as control by securing and documenting employee’s competency
- as an aid to learn about or be introduced to equipment, guidelines, etc.
- as a means for the employer to transfer onto employees the responsibility of professionals to remain up-to-date [11].

The quality of evidence regarding internet-based learning is weak, and therefore its effect on guideline adherence is unknown [52]. We believe that e-learning courses are suitable to serve as an introduction in the process of implementing a new guideline, but is probably insufficient alone. The result of this study supports the finding of other studies that suggests that training is of importance to prehospital personnel [50, 52]. It seems that several services are in the process of implementing e-learning, but that e-learning played a minor role when considering implementation of this guideline.

## Limitations

There are several limitations to this study. The study does not distinguish between the effect of passive dissemination measures, the e-learning course, and implementation measures conducted locally. The study was not designed to assess adherence to the guideline, as the main focus was whether or not the guideline was implemented in the EMS. The response rate was low, but the figure was based on the number of employed prehospital personnel from Statistics Norway. A large number of personnel reported in the statistics are probably not in active service, or are in reduced positions, as a result of the very liberal criteria required to be counted in the register. The response rate affects the external validity and enables possible selection bias.

## Conclusion

To raise awareness and implement new guidelines among health care personnel is in general a challenging task. This guideline was developed based on perceived needs among care providers. Although the guideline was not authorized or compulsory at a national level, one-half of the respondents with knowledge of the guideline reported it as having been implemented. Implementation through passive dissemination was therefore possible, and we speculate that the background of the guideline facilitated its implementation. Regarding the E-learning course, there is uncertainty associated with its effect on implementation in this instance. Concerning the rather low response rate, surveys seems to be an unsuitable method to gather information regarding important research questions unless greater awareness among prehospital personnel for the shared responsibility to bring our discipline forwards.

## Additional file

Additional file 1: Questions from the survey. (DOCX 14 kb)

## Data Availability

The dataset used and analysed during this study is available from the corresponding author on reasonable request.

## Abbreviations

AGREE: Appraisal of Guidelines for Research and Evaluation
ED: Emergency Department
EMS: Emergency Medical Services
NAKOS: Norwegian National Advisory Unit on Prehospital Emergency Medicine
NKT-T: Norwegian National Advisory Unit on Trauma
NOKC: Norwegian Knowledge Centre for the Health Services
