# Supplementary material for: Implementation of new guidelines in the prehospital services: a nationwide survey of Norway
Source: Scand J Trauma Resusc Emerg Med. 2019 Aug 29;27:83. doi: 10.1186/s13049-019-0660-0 (PMC6716817; doi:10.1186/s13049-019-0660-0)
Supplement: Supplementary file 1 — Questions from the survey. (DOCX 14 kb) [file 13049_2019_660_MOESM1_ESM.docx]

**Additional file 1 Questions from the survey**

**Have you heard about the national guideline “Stabilizing the spine from the scene of accident to clarification”? Alternatives: Yes/No**

**Is the guideline going to be used in the service where you work? Alternatives:**

**Yes/No/I do not know**

**Is the guideline “Stabilizing the spine” being used in the service where you work?**

**Alternatives: Yes/No/I do not know**

**Have you executed the guideline “Stabilizing the spine” in your work?**

**Alternatives: Yes/No**

**If no, why not (several answers possible)?**

**The guideline is not approved by the employer**

**I have not been trained properly to use the guideline**

**I do not have confidence in the guideline**

**I do not understand the guideline**

**The (written) guideline is not available when I am responding to a call**

**I have not responded to calls where the guideline was needed**

**Other**

**Have you received feedback in the ED when delivering patients treated according to the guideline? Alternatives: Yes/No**

**What** **kind of feedback did you receive? Open question**

**Is the guideline of practical benefit for you when assessing whether the patient should be stabilized? Alternatives: Yes/No**

**Is the flowchart that follows the guideline easy to understand? Alternatives:Yes/No**

**Do you bring the flowchart when on duty? Alternatives: Yes/No**

**Is the flowchart that follows the guideline easy to use when you are working at the scene of an accident? Yes/No/I do not know**

**Do you have any comments on the guideline or the flowchart? Open question**

**Does your service use the NAKOS web portal for certifications and courses?**

**Alternatives: Yes/No**

**Have you completed the e-learning course “Stabilizing the spine" in the NAKOS web portal? Alternatives: Yes/No**
